# Supplementary material for: The genome of the soybean gall midge (Resseliella maxima)
Source: G3 (Bethesda). 2023 Mar 2;13(4):jkad046. doi: 10.1093/g3journal/jkad046 (PMC10085792; doi:10.1093/g3journal/jkad046)
Supplement: jkad046_Supplementary_Data [file jkad046_supplementary_data.zip › Supplemental Material Legends.docx]

**Supplemental Data**

**Supplementary File 1.** Sequencing run statistics table

| **Seq Run** | **num_seqs** | **sum_len** | **avg_len** | **N50** | **Q20(%)** | **Q30(%)** |
| --- | --- | --- | --- | --- | --- | --- |
| Run 1 (R9.4.1) | 2,016,993 | 4,650,614,893 | 2,305 | 3,269 | 69.16 | 36.24 |
| Run 2 (R9.4.1) | 806,605 | 1,505,157,896 | 1,866 | 2,767 | 59.28 | 28.98 |
| Run 3 (R10.4.1) | 3,213,250 | 7,530,779,825 | 2,343 | 3,847 | 83.73 | 66.9 |

**Supplementary File 2.**

Genome-wide methylation figures

**Supplementary File 3.**

Repeat information

**Supplementary File 4.**

Protein annotation data

**Supplementary File 5.**

Mitogenome gene order comparison between *R. maxima* and *O. oryzae.* Maximum likelihood phylogenetic tree, accession numbers, trimmed alignments, and newick file of Cecidomyiidae cytochrome oxidase I (COX1) sequences from NCBI.

**Supplementary File 6.**

Kraken2 output identifying all read hits to *Wolbachia* species.

**Supplementary File 7.**

Detailed computational methods
