# Supplementary figures and images for: The genome of the soybean gall midge (Resseliella maxima)
Source: G3 (Bethesda). 2023 Mar 2;13(4):jkad046. doi: 10.1093/g3journal/jkad046 (PMC10085792; doi:10.1093/g3journal/jkad046)

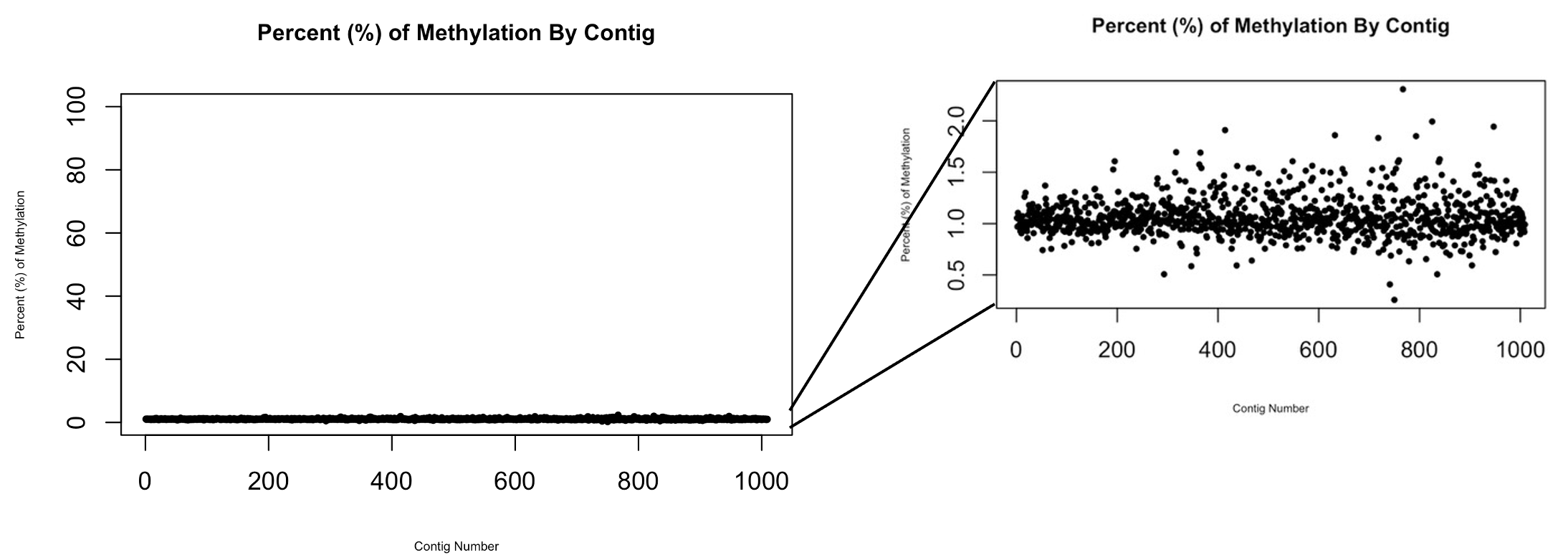

Supplement: jkad046_Supplementary_Data [file jkad046_supplementary_data.zip › Supp File 2.png]
