# Supplementary material for: The genome of the soybean gall midge (Resseliella maxima)
Source: G3 (Bethesda). 2023 Mar 2;13(4):jkad046. doi: 10.1093/g3journal/jkad046 (PMC10085792; doi:10.1093/g3journal/jkad046)
Supplement: jkad046_Supplementary_Data [file jkad046_supplementary_data.zip › Supp File 5.mitogenome-comparison-and-treefile.zip!/mitogenome-comparison-and-treefile/mitogenome_tree_color_coded.pdf]

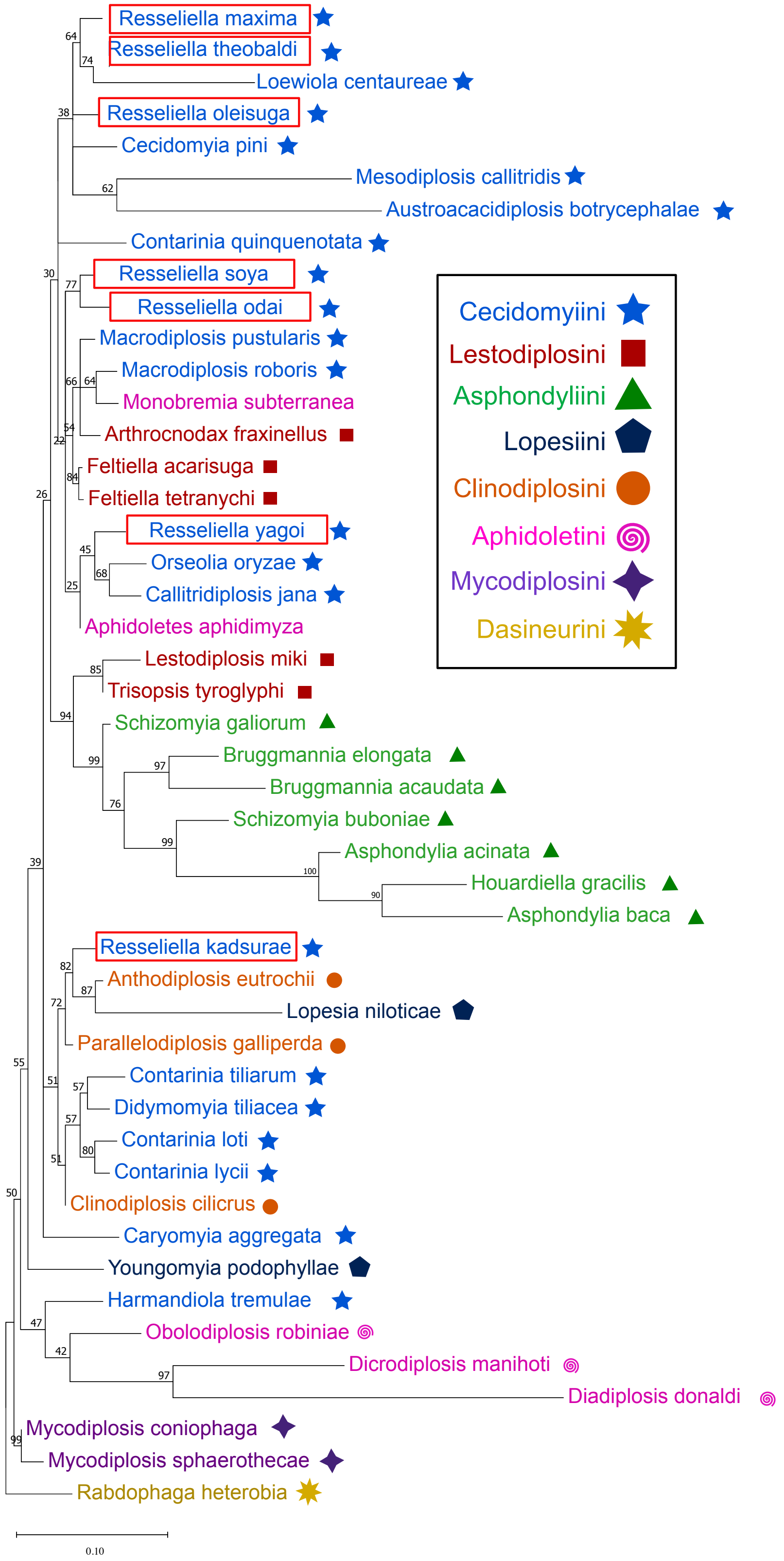

Figure. Maximum likelihood phylogenetic tree of Cecidomyiidae cytochrome oxidase I (COX1) sequences from NCBI. Node labels indicate bootstrap support. Red boxes indicate Resseliella species
