# Supplementary material for: The genome of the soybean gall midge (Resseliella maxima)
Source: G3 (Bethesda). 2023 Mar 2;13(4):jkad046. doi: 10.1093/g3journal/jkad046 (PMC10085792; doi:10.1093/g3journal/jkad046)
Supplement: jkad046_Supplementary_Data [file jkad046_supplementary_data.zip › Supp File 5.mitogenome-comparison-and-treefile.zip!/mitogenome-comparison-and-treefile/Supp 5 mito_compare.pdf]

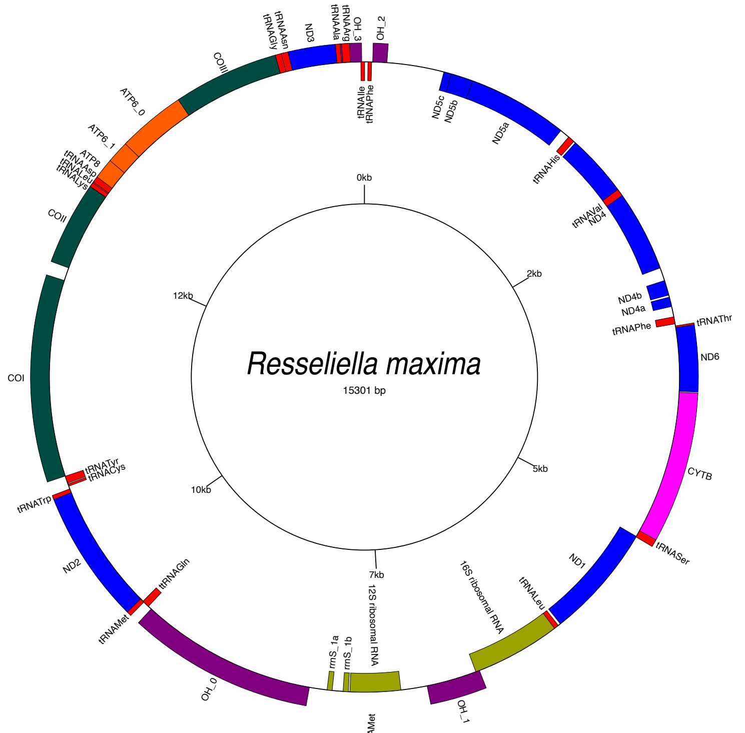
Resseliella maxima
15301 bp
0kb
2kb
5kb
7kb
10kb
12kb
OH_2
OH_3
tRNAArg
tRNAAla
ND3
tRNAAsn
tRNAGly
COIII
ATP6_0
ATP6_1
ATP8
tRNAAsp
tRNALeu
tRNALys
COII
COI
tRNATyr
tRNACys
tRNATrp
ND2
tRNAGln
tRNAMet
OH_0
rrnS_1a
rrnS_1b
12S ribosomal RNA
OH_1
16S ribosomal RNA
tRNALeu
ND1
tRNASer
CYTB
ND6
tRNAThr
tRNAPhe
ND4a
ND4b
ND4
tRNAVal
tRNAHis
ND5a
ND5b
ND5c
tRNAIle
tRNAPhe

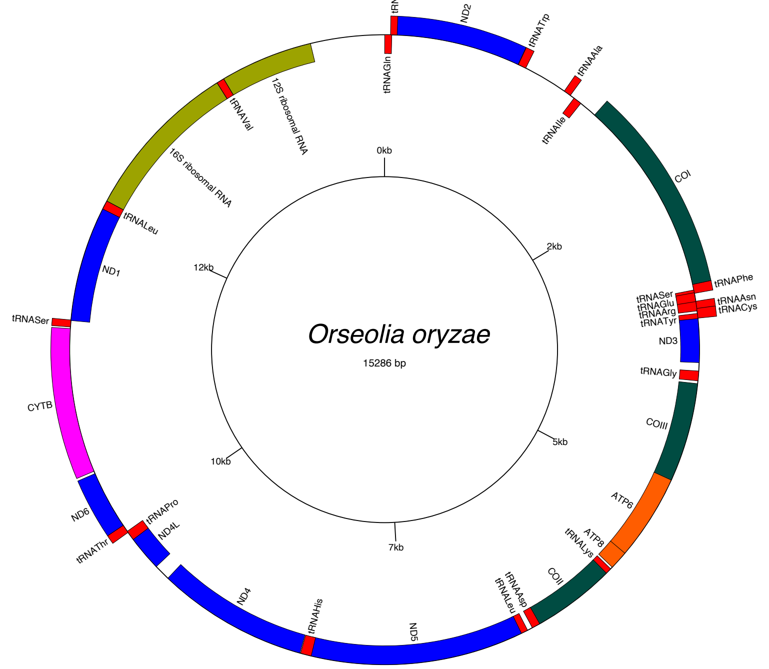
Orseolia oryzae
15286 bp
0kb
2kb
5kb
7kb
10kb
12kb
tRNAMet
ND2
tRNATrp
tRNAAla
tRNAIle
COI
tRNAPhe
tRNAAsn
tRNACys
tRNASer
tRNAGlu
tRNAArg
tRNATyr
ND3
tRNAGly
COIII
ATP6
ATP8
tRNALys
COII
tRNAAsp
tRNALeu
ND5
tRNAHis
ND4
ND4L
tRNAPro
tRNAThr
ND6
CYTB
tRNASer
ND1
tRNALeu
16S ribosomal RNA
tRNAVal
12S ribosomal RNA
tRNAGln
