# Supplementary material for: The genome of the soybean gall midge (Resseliella maxima)
Source: G3 (Bethesda). 2023 Mar 2;13(4):jkad046. doi: 10.1093/g3journal/jkad046 (PMC10085792; doi:10.1093/g3journal/jkad046)
Supplement: jkad046_Supplementary_Data [file jkad046_supplementary_data.zip › Supp File 7.html.zip!/Supp 6.html]

Soybean Gall Midge Genome Notebook


Code 

- Show All Code
- Hide All Code
- Download Rmd

# Soybean Gall Midge Genome Notebook

#### Chris Faulk


```
library(tidyverse)
```


Soybean Gall Midge.

# Sequencing and Basecalling

Sequencing was performed on two flow cells, an R9.4.1 and an R10.4.1.
Each cell was loaded and run for 24 hours, then nuclease flushed and
reloaded an additional 24 hours for a total of 72 hours. Post-hoc
basecalling was performed using guppy with the following parameters to
include modified basecalling at CpG sites.

```
guppy_basecaller --input_path . -r -s sgm-output/ -c dna_r10.4.1_e8.2_400bps_modbases_5mc_cg_sup.cfg --bam_out --compress_fastq --nested_output_folder --min_qscore 10`
```

# Full Assembly

### Flye `--asm-coverage 128`

Assemblies were compared with the full read set and a subset of
>3400 bp reads. The full read set yielded better assemblies as
measured by BUSCO and was used for all further processing.

### Install

Flye 2.9.1-b1780 was installed from github via conda

```
conda env create -n flye -c bioconda flye=2.9.1
conda activate flye
```

### Run

The `--asm-coverage` option limits the minimum required
coverage for the initial disjointig extension state, a memory bottleneck
in the program. Setting `--genome-size` also reduces memory
usage.

```
flye --nano-hq sgm-total.fastq --out-dir flye_results_sgmtotal --genome-size 200m --asm-coverage 128 --threads 23 --no-alt-contigs
```

The run completed in ~3 hours on a 12 core AMD 5900.

### BUSCO

BUSCO scores are used to assess assembly quality without reliance on
inherent non-biologically relevant characteristics of the assembly. It
identifies phylum-unique genes within the assembly. So, this assembly
should contain a high number of the genes unique to Diptera

#### Install

The command line package `busco` v5.4.2 was installed in a
`mamba` virtual environment on via
`mamba install -c bioconda -c conda-forge busco=5.4.2`.
*Note: the mamba documentation says you should still use conda to
activate and deactivate*. Full environment specs in
`busco_conda_env.txt`.

#### Run

`busco` in genome mode was run in a mamba (conda) virtual
environment, taking approximately 15 minutes.

#### Diptera lineage

`busco -i assembly.fasta -o busco_output_draft -m genome --lineage diptera -c 24`

Results via auto-generated short\_summary text file:

```
# BUSCO version is: 5.4.2 
# The lineage dataset is: diptera_odb10 (Creation date: 2020-08-05, number of genomes: 56, number of BUSCOs: 3285)
# Summarized benchmarking in BUSCO notation for file /home/cfaulk/Desktop/ont-sequencing_runs/soybean_gall_midge/sgm-analysis/flye_results_sgmtotal/medaka-results/consensus.fasta
# BUSCO was run in mode: euk_genome_met
# Gene predictor used: metaeuk

    ***** Results: *****
    C:87.9%[S:85.1%,D:2.8%],F:1.9%,M:10.2%,n:3285      
    2886    Complete BUSCOs (C)            
    2795    Complete and single-copy BUSCOs (S)    
    91  Complete and duplicated BUSCOs (D)     
    63  Fragmented BUSCOs (F)              
    336 Missing BUSCOs (M)             
    3285    Total BUSCO groups searched        

Assembly Statistics:
    2613    Number of scaffolds
    2613    Number of contigs
    211232549   Total length
    0.000%  Percent gaps
    698 KB  Scaffold N50
    698 KB  Contigs N50

Dependencies and versions:
    hmmsearch: 3.1
    bbtools: 39.01
    metaeuk: 5.34c21f2
    busco: 5.4.2
```

### Polish

Medaka v1.7.2 was used to polish the assembly.

```
conda env create -n medaka -c bioconda flye=1.7.2
conda activate medaka

#Note that r941_min_sup_g507 model was used because some of the data came from an r9 flow cell.
medaka_consensus -b 100 -i sgm-total.fasq -d assembly.fasta -o medaka-results -t 24 -m r941_min_sup_g507
```

##### BUSCO

`/home/cfaulk/miniconda3/bin/busco -i medaka-results/consensus.fasta -o sgm-total-medaka-busco -m genome -l diptera -c 24`

Result:
`C:87.9%[S:85.1%,D:2.8%],F:1.9%,M:10.2%,n:3285`

### Purge haplotigs

Skipped purge\_haplotigs, since separate peaks were not obvious,
indicating lack of demonstrated haplotig presence.

```
minimap2 -t 24 -ax map-ont consensus.fasta ../sgm-total.fastq | samtools sort -m 1G -o aligned.bam -T tmp.ali
purge_haplotigs  hist  -b aligned.bam  -g consensus.fasta  -t 24
```

## BLAST for contaminants

Blast was performed and provided to Blobtools as input. A local
installation of blast++ and the non-redundant (nr) database were used to
identify contigs.

`blastn -query consensus.fasta -task megablast -db ~/Desktop/genomes/nt/nt -outfmt '6 qseqid staxids bitscore std sscinames sskingdoms stitle' -culling_limit 10 -num_threads 23 -evalue 1e-3 -out consensus.fasta.vs.nt.cul5.1e3.megablast.out`

Megablast results are saved as
`consensus.fasta.vs.nt.cul5.1e3.megablast.out`, assembly as
`consensus.fasta`, coverage information as
`consensus.coverage.txt`, and BUSCO results as
`full_table.tsv`. These files are ready for Blobtoolkit
input.

## Contaminant searching with Blobtoolkit

### Pip install

Install
instructions Installs version 3.2.6

```
pip install blobtoolkit
pip install fastjsonschema
```

### Generate coverage file

```
minimap2 -ax map-ont -t 24 consensus.fasta sgm-total.fastq | samtools sort -@16 -O BAM -o consensus.sorted.bam -
```

Generate coverage .txt
`samtools coverage consensus.sorted.bam > consensus.coverage.txt`.

### Create blobdir

blobtoolkit v3.2.6

Download taxdump from NCBI,
unzip, and moved all the resulting `.dmp` files to the
directory `taxdump`.

Create the blobdir and load it with files.

```
blobtools create --fasta consensus.fasta --meta data/midge.yaml --taxid 2494512 --taxdump taxdump/ datasets/midge
#Add BUSCO
blobtools add --busco full_table.tsv datasets/midge
#Add coverage
blobtools add --text consensus.coverage.txt --text-header --text-cols '#rname=identifier,meandepth=midge_reads_cov' datasets/midge
#Fix axes
blobtools add --key plot.y=midge_reads_cov datasets/midge
```

### View

`blobtools view --local --interactive --ports 4000-4999 datasets/midge`

## Decontamination

We filter on the following criteria to remove short and low coverage
contigs. These filter settings result in no loss of full length
BUSCOs.

Remove length less than 8000 bp.  
Remove coverage less than 20X and greater than 200X.  
Filtered contigs are exported in .csv format from blobtools

2613 Original Contigs  
1009 Remaining Contigs saved as `1009.clean.contigs.csv`

### Filter assembly

Use seqkit to filter the consensus assembly to only the 1009 contigs
from decontamination.

`seqkit grep -f 1009.contigs.id.txt ../blobtools-out/consensus.fasta -o clean.consensus.fasta`

## Final BUSCO

`busco -i clean.consensus.fasta -o busco-cleanconsensus -m genome -l ~/Desktop/genomes/busco_downloads/lineages/diptera_odb10 -c 24`

Result:

```
    ***** Results: *****

    C:87.8%[S:85.2%,D:2.6%],F:1.9%,M:10.3%,n:3285      
    2886    Complete BUSCOs (C)            
    2800    Complete and single-copy BUSCOs (S)    
    86  Complete and duplicated BUSCOs (D)     
    63  Fragmented BUSCOs (F)              
    336 Missing BUSCOs (M)             
    3285    Total BUSCO groups searched
```

## Methylation

Use the unmapped .bam files generated during the guppy basecalling.
Cat together modbams for each flow cell directory:
`samtools cat -o sgm-mod.unmapped.bam bug1/*.bam bug2/*.bam bug3/*.bam`

### Map Unmapped Bams.

Map, convert and sort all in one command. The `-T Mm,Ml`
flag carries over the modifications from bam to fastq. In newer bams the
flags are `-T MM, ML`. The `-y` flag copies input
fastq commands to output.
`samtools fastq -T Mm,Ml sgm-mod.unmapped.bam | minimap2 -y -ax map-ont ../final_assembly/clean.consensus.fasta - -t 24 | samtools view -u - | samtools sort -@ 24 -o sgm.modmapped.bam; samtools index sgm.modmapped.bam -@ 24`

Convert to bed format. The `-d` flag limits depth to save
memory. The aggregate flag adds together bases on either strand and
creates and aggregate.bed file. The `-cpg` flag limits to cpg
sites.
`modbam2bed -m 5mC -t 23 -e -d 300 -p sgm.modmapped --aggregate --cpg clean.consensus.fasta sgm.modmapped.bam > sgm.modmapped.bed`

Assess global DNA methylation as a percentage by adding up all
canonical (column 12) and modified (column 13) cytosine base calls at
CpG sites. Divide the modified by the total and print.

`awk '$5>0 {can+=$12; mod+=$13} END{print 100*(mod/(can+mod))}' sgm.mapped.bed`

### Global Methylation

1.07294 is the global methylation in Soybean Gall Midge

## Mosdepth Assembly Stats

Depth statistics generated with mosdepth

### Install

Downloaded v0.3.3 binary from Github release
page.

### Run

1. Overall depth stats:
   `mosdepth -n --fast-mode -t 8 sgm.modmapped ../methylation/sgm.modmapped.bam`

   `chrom length bases mean min max`

   `total 206036186 13366775054 64.88 0 43586`
2. For 500 bp windows:
   `mosdepth -n --fast-mode --by 500 -t 8 500bpwindow.sgm.modmapped ../methylation/sgm.modmapped.bam`

   `chrom length bases mean min max`

   `total_region 206036186 13366775054 64.88 0 43586`

# Repeats

## Repeat Identification

Since annotated repeats in insect genomes are sparse, repeat
identification requires two stages. First *de novo* repeat
identification was performed with RepeatModler2.
Second, the libraries generated with RepeatModeler2 were used as input
to RepeatMasker to create a
complete genome annotation of repeats with existing names for classes,
families, and subfamilies where known and novel IDs for previously
unknown families.

RepeatModeler was run to identify repeat content. Takes about 24
hours on a 300 Mb insect genome.

1. First build database from consensus fasta.  
   `/usr/local/RepeatModeler-2.0.2a/BuildDatabase -name "sgm-genome" ../final-assembly/consensus.clean.fasta`
2. Run RepeatModeler2.  
   `nohup <RepeatModelerPath>/RepeatModeler -database sgm-genome -pa 14 -LTRStruct >& run.out &`
3. RepeatMasker was then run to determine genomic repeat content
   specifying the custom library created with RepeatModeler.  
   `RepeatMasker -pa 14 -s -xsmall -lib sgm-genome-families.fa ../final-assembly/consensus.clean.fasta`

Comparison to other midge genomes was performed using Dfam v3.6 and
by ab initio identification using the same RepeatModeler2 pipeline as
above.

# GeMoMa Gene Annotation

Wiki

### Install

Install depencency `mmseq`:

`conda install -c conda-forge -c bioconda mmseqs2`

Installed version 1.9 by downloading `.zip` file from website

### vs. Drosophila melanogaster (dm6) and Swede midge (*Contarinia nasturtii*)

Two annotations of diptera were used as a reference based on close
phylogenetic proximity to the soybean gall midge and annotation
availability. FASTA and GFF files were downloaded from NCBI’s Assembly
page. **Note:** A hardmasked version of the genome (repeats
replaced with N’s) was used for input to eliminate transposon derived
genes.

`java -Xmx50g -jar gemoma-java/GeMoMa-1.9.jar CLI GeMoMaPipeline threads=23 outdir=combined_Dmel_swedemidge GeMoMa.Score=ReAlign AnnotationFinalizer.r=NO o=true t=clean.consensus.hardmasked.fasta s=own i=swede_gall_midge a=GCF_009176525.2_AAFC_CNas_1.1_genomic.gff g=GCF_009176525.2_AAFC_CNas_1.1_genomic.fna s=own i=D_mel g=GCA_000001215.4_Release_6_plus_ISO1_MT_genomic.fna a=GCA_000001215.4_Release_6_plus_ISO1_MT_genomic.gff`

BUSCO was used to assess completeness in protein mode:

`busco -i predicted_proteins.fasta -o busco -m protein -l diptera -c 24`

```
    --------------------------------------------------
    |Results from dataset diptera_odb10               |
    --------------------------------------------------
    |C:89.9%[S:85.5%,D:4.4%],F:0.7%,M:9.4%,n:3285     |
    |2952   Complete BUSCOs (C)                       |
    |2808   Complete and single-copy BUSCOs (S)       |
    |144    Complete and duplicated BUSCOs (D)        |
    |24 Fragmented BUSCOs (F)                     |
    |309    Missing BUSCOs (M)                        |
    |3285   Total BUSCO groups searched               |
    --------------------------------------------------
2023-01-05 18:39:09 INFO:   BUSCO analysis done. Total running time: 110 seconds
2023-01-05 18:39:09 INFO:   Results written in /home/cfaulk/Desktop/ont-sequencing_runs/soybean_gall_midge/sgm-analysis/gemoma-out/combined_Dmel_swedemidge/busco-combined-protein
```

# Mitochondrial genome

Total reads were first blasted by mtblaster (https://github.com/nidafra92/squirrel-project/blob/master/mtblaster.py))
using the Orseolia oryzae mitogenome (KM888183) to select for reads with
high identity to a gall midge mitochondria sequence. We blasted against
reads only from the R10 flowcell to ensure higher accuracy. Next,
resulting reads were filtered by nanofilt (https://github.com/wdecoster/nanofilt))
to keep only reads above 15 kbp in length and average Q-score above 30.
Finally, flye was used to perform mitogenome assembly (Kolmogorov et al.
2020). A single circular contig was recovered with 785X coverage. The
assembly was polished using four rounds of racon (https://github.com/lbcb-sci/racon) followed by medaka
with the same parameters as the nuclear assembly.

1. Blast against reads from the R10 flow cell:
   `mtblaster.py sgm-bug3.fastq Orseolia_oryzae-KM888183.1.fasta`
2. Filter reads higher than 15 kb with quality >Q30.
   `nanofilt -l 15000 -q 30 reads_w_hits.fastq > reads_w_hits15kq30.fastq`
3. Run flye to assemble genome using the asm parameter.
   `flye --nano-hq reads_w_hits15kq30.fastq --out-dir flye-mitogenome-highasm --genome-size 16k --threads 24 --asm 256`
4. Run 4 rounds of Racon polishing,
   `minimap2 -ax map-ont assembly.fasta ../reads_w_hits15kq30.fastq > assembly.sam`

   `racon -t 24 ../reads_w_hits15kq30.fastq assembly.sam assembly.fasta > assembly.racon1.fasta`
5. Final polish with medaka.
   `medaka_consensus -i ../reads_w_hits15kq30.fastq -d assembly.racon4.fasta -o medaka-results/ -t 23 -m r1041_e82_400bps_sup_g615`
6. Save `consensus.fasta` as
   `sgm_mitogenome_consensus.fasta`

# Wolbachia detection

Collect panel of 9 genomes into a single fasta file and match against
the total SGM read set. Identify any resulting matches against the
kracken2 standard database. The genomes were downloaded from NCBI and
are listed in the manuscript.

1. Match reads to Wolbachia genomes.
   `minimap2 -ax map-ont wolbachia-panel/wolbachia-panel.fna ../sgm-total.fastq.gz -t 23 > wolbachia-panel.vs.sgm-total.sam`
2. Sort and convert to bam format.
   `samtools sort -@24 -O BAM -o sgm-total.vs.wolbachia-panel.sorted.bam wolbachia-panel.vs.sgm-total.sam`
3. Filter alignment file for only mapped reads.
   `samtools fasta -F 4 sgm-total.vs.wolbachia-panel.sorted.bam > sgm-total.vs.wolbachia-panel.sorted.hits.bam`
4. Remove duplicates.
   `awk '/^>/{f=!d[$1];d[$1]=1}f' sgm-total.vs.wolbachia-panel.sorted.hits.fasta > sgm-total.vs.wolbachia-panel.sorted.nodups.fasta`
5. Run through Kraken2.
   `kraken2 --db /mnt/data16Tb/kraken_db/k2_standard_16gb_20220607 --threads 23 --use-names --report sgm.vs.wolbachia-panel.report.txt --output sgm.vs.wolbachia-panel.txt sgm-total.vs.wolbachia-panel.sorted.nodups.fasta`
6. Run Pavian from inside R to generate graphics.
   `pavian::runApp(port=5000)`

# NCBI Submission

Link

Used command line to upload folder:

1. Navigate to the source folder where the files for submission
   are;
2. Establish an FTP connection using the credentials below: Address:
   `ftp-private.ncbi.nlm.nih.gov` Username: `subftp`
   Password: `<sent via email>`
3. Navigate to your account folder: From the command line use the ‘cd’
   command: `cd uploads/<folder_sent_by_email>`

When using a GUI FTP client (eg: Filezilla, NcFTP, Cyberduck, etc.),
after you’ve connected to the FTP server, paste your account folder
(`uploads/<folder_sent_by_email>`) into the “Remote
Site” or “Remote Directory” box on the interface and press “Enter”.

Then you will be able to create the data sub-directory for your
submission. Until you do this, you will see a message stating “550 /:
Permission denied” or “Failed to read the directory listing”. We prevent
directory listing in the default sign in folder for security
reasons.

Create a subfolder (required!) with a meaningful name. Navigate to
the target folder you just created. Copy your files into the target
folder:
`<mitogenome>.fa.gz <nuclear_genome>.fa.gz`

Press “New submission” in browser.
